# Lightweight Deep Learning Framework for Accurate Detection of Sports-Related Bone Fractures

**DOI:** 10.3390/diagnostics15030271

**Published:** 2025-01-23

**Authors:** Akmalbek Abdusalomov, Sanjar Mirzakhalilov, Sabina Umirzakova, Otabek Ismailov, Djamshid Sultanov, Rashid Nasimov, Young-Im Cho

**Affiliations:** 1 Department of Computer Engineering, Gachon University, Seongnam 13120, Republic of Korea; akmaljon@gachon.ac.kr; 2 Department of Computer Systems, Tashkent University of Information Technologies Named After Muhammad Al-Khwarizmi, Tashkent 100200, Uzbekistan; mirzaxalilov86@tuit.uz (S.M.); vice-rector@tuit.uz (D.S.); 3 International School of Finance Technology and Science, ISFT Institute, Tashkent 100200, Uzbekistan; ismoilovotabek2023@gmail.com; 4 Department of Artificial Intelligence, Tashkent State University of Economics, Tashkent 100066, Uzbekistan; rashid.nasimov@tsue.uz; 5 Department of International Scientific Journals and Rankings, Alfraganus University, Tashkent 100190, Uzbekistan

**Keywords:** bone fracture detection, lightweight deep learning, sports medicine, real-time diagnostics, radiographic analysis

## Abstract

**Background/Objectives:** Sports-related bone fractures are a common challenge in sports medicine, requiring accurate and timely diagnosis to prevent long-term complications and enable effective treatment. Conventional diagnostic methods often rely on manual interpretation, which is prone to errors and inefficiencies, particularly for subtle and localized fractures. This study aims to develop a lightweight and efficient deep learning-based framework to improve the accuracy and computational efficiency of fracture detection, tailored to the needs of sports medicine. **Methods:** We proposed a novel fracture detection framework based on the DenseNet121 architecture, incorporating modifications to the initial convolutional block and final layers for optimized feature extraction. Additionally, a Canny edge detector was integrated to enhance the model ability to detect localized structural discontinuities. A custom-curated dataset of radiographic images focused on common sports-related fractures was used, with preprocessing techniques such as contrast enhancement, normalization, and data augmentation applied to ensure robust model performance. The model was evaluated against state-of-the-art methods using metrics such as accuracy, recall, precision, and computational complexity. **Results:** The proposed model achieved a state-of-the-art accuracy of 90.3%, surpassing benchmarks like ResNet-50, VGG-16, and EfficientNet-B0. It demonstrated superior sensitivity (recall: 0.89) and specificity (precision: 0.875) while maintaining the lowest computational complexity (FLOPs: 0.54 G, Params: 14.78 M). These results highlight its suitability for real-time clinical deployment. **Conclusions:** The proposed lightweight framework offers a scalable, accurate, and efficient solution for fracture detection, addressing critical challenges in sports medicine. By enabling rapid and reliable diagnostics, it has the potential to improve clinical workflows and outcomes for athletes. Future work will focus on expanding the model applications to other imaging modalities and fracture types.

## 1. Introduction

**Figure 1:**
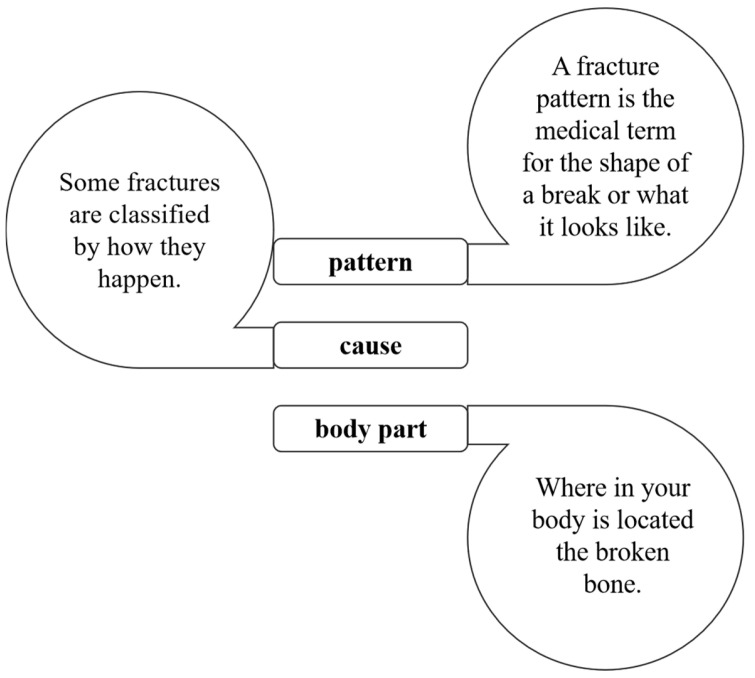
Types of bone fractures. Illustration depicting various bone fracture patterns, including transverse, spiral, greenstick, oblique, and compression fractures, commonly encountered in sports-related injuries.

Bone fractures, commonly known as broken bones, represent a significant concern in sports medicine due to their prevalence among athletes engaged in high-impact and repetitive physical activities [1] (Figure 1). These injuries not only compromise the health and professional longevity of athletes but also impose substantial medical and economic burdens on healthcare systems worldwide [2]. Early and accurate detection of bone fractures is critical for effective treatment, minimizing recovery time, and preventing long-term complications, such as functional impairments or the necessity for invasive surgical interventions [3]. Despite advancements in medical imaging technologies, such as X-rays, CT scans, and MRI, the accurate and efficient diagnosis of fractures remains a challenge, particularly in high-performance sports contexts where rapid assessments are crucial [4]. Current diagnostic practices rely heavily on manual interpretation by radiologists, which is subject to human error and variability in clinical expertise [5]. Furthermore, subtle or minor fractures, which are often precursors to severe injuries, may be overlooked, emphasizing the need for enhanced diagnostic methods.

The integration of artificial intelligence (AI) into medical imaging has demonstrated significant potential for automating and improving diagnostic accuracy across various clinical domains [6]. Deep learning models, particularly convolutional neural networks (CNNs), have emerged as powerful tools capable of extracting intricate patterns and features from complex medical images [7]. Among these, DenseNet architectures have gained prominence due to their efficient parameter utilization and superior performance in image classification tasks [8]. However, challenges remain in optimizing these models for detecting small and localized features, such as those indicative of bone fractures. In this study, we address these challenges by proposing an innovative deep learning-based framework tailored for fracture detection in athletes. Building upon the DenseNet121 architecture, we introduce novel enhancements, including the integration of a Canny edge detector and modifications to key network layers. The edge detector enables the model to focus on localized structural discontinuities characteristic of fractures, while architectural adjustments improve the model sensitivity and specificity. Our approach is further supported by a custom-curated dataset of medical images, focusing on fractures commonly encountered in athletic activities. This study presents a novel deep learning-based framework tailored for detecting sports-related bone fractures, addressing critical challenges in medical image analysis and sports medicine. Our contributions are summarized as follows:

- We integrate the Canny edge detector within our model architecture to enhance its ability to identify subtle structural discontinuities indicative of bone fractures. This addition significantly improves the model performance in detecting minor and complex fractures.
- Building upon the DenseNet121 architecture, we propose modifications to the initial convolutional block and the final layers, optimizing the model for better feature learning. These changes ensure improved sensitivity and specificity, critical for accurately diagnosing fractures.
- Our proposed model achieves a state-of-the-art accuracy of 90.3%, recall of 0.89, and precision of 0.875. These results surpass existing models, such as ResNet-50, VGG-16, and EfficientNet-B0, demonstrating the effectiveness of our approach.
- With a lightweight architecture requiring only 14.78 million parameters and 0.54 GFLOPs, the model is computationally efficient, making it highly suitable for real-time clinical use in resource-constrained environments.
- A custom-curated dataset focused on fractures prevalent among athletes was utilized, supported by preprocessing techniques like contrast enhancement and data augmentation, ensuring the robustness and generalizability of the model.
- The proposed framework addresses the critical need for rapid and precise diagnosis in clinical settings, potentially reducing recovery times and minimizing invasive interventions for athletes, thereby improving patient outcomes.

This research aims to advance the state of the art in medical image analysis by presenting a robust and efficient model that not only achieves superior diagnostic accuracy but also operates with reduced computational complexity. By facilitating early and precise fracture detection, the proposed methodology has the potential to significantly improve clinical outcomes for athletes and establish a new benchmark in sports injury diagnostics.

## 2. Related Works

The intersection of artificial intelligence (AI) and medical imaging has garnered substantial attention in recent years, with numerous studies exploring deep learning techniques for the detection and classification of bone fractures [9]. Conventional approaches to fracture diagnosis primarily rely on manual interpretation of radiographic images by trained radiologists [10]. However, these methods are inherently subjective and prone to variability, often leading to delayed or inaccurate diagnoses, particularly in cases involving subtle or complex fractures. Recent advancements in convolutional neural networks (CNNs) have demonstrated transformative potential in automating diagnostic tasks in medical imaging [11]. Pioneering architectures such as ResNet, VGG, and DenseNet have achieved remarkable success in classifying medical images, owing to their ability to extract intricate patterns from high-dimensional data [12]. For example, ResNet skip connections have addressed the vanishing gradient problem, enhancing the depth and performance of CNNs in image analysis tasks. Similarly, DenseNet densely connected layers have facilitated efficient parameter utilization and improved feature propagation, making it a preferred choice for medical applications.

In the specific domain of fracture detection, several studies have leveraged CNN-based models. Lui et al. [13] proposed a multi-scale CNN architecture for detecting fractures in radiographic images, achieving notable accuracy improvements. Similarly, Tieu et al. [14] integrated attention mechanisms into a ResNet-based framework to enhance the detection of subtle fractures. Ju et al. [15] focus on improving pediatric wrist fracture detection using the YOLOv8 algorithm. Kassem et al. [16] introduce an accurate computer-aided diagnosis (CAD) system based on deep learning for detecting pelvis fractures, addressing the shortage of experienced radiologists. Karanam et al. [17] introduce the Finite Beta Gaussian Mixture Model (FBGMM) for classifying bone fractures from X-ray images. The model demonstrates robust performance in both binary and multiclass fracture classification, evaluated using performance metrics and a confusion matrix. In the study by Zou et al. [18], the YOLOv7-ATT model conducts a comparative analysis of one-stage and two-stage deep learning architectures for bone fracture detection, focusing on four fracture morphologies: angle fractures, normal fractures, line fractures, and messed-up angle fractures. To improve accuracy and timeliness in diagnosis, Medaramatla et al. [19] developed a hybrid deep learning model that combines YOLO NAS, EfficientDet, and DETR3 algorithms, known for their precise object detection capabilities. Yıldız et al. [20] focus on developing a deep learning model for detecting and localizing proximal femur fractures from hip radiographs, addressing the increasing global incidence of hip fractures. The authors enhanced the diagnostic capabilities of their model by extending the VarifocalNet Feature Pyramid Network (FPN) to better identify and classify fracture types. Saad et al.’s [21] study highlights the development of a CNN model for automated and precise detection of bone fractures in X-ray images, addressing the critical need for timely and accurate diagnosis in clinical imaging. Chien et al. [22] introduce the application of YOLOv9, the latest in the YOLO series, for fracture detection in X-ray images to assist radiologists and surgeons. These studies underscore the importance of architectural innovations in addressing the challenges of medical imaging, such as small region-of-interest detection and the variability of image quality. Despite these advancements, existing models often struggle with detecting minor or localized fractures, particularly in medical images with high noise levels or varying resolutions [23]. Additionally, many models are computationally intensive, posing challenges for real-time deployment in clinical environments [24]. Addressing these limitations requires a balance between diagnostic accuracy and computational efficiency.

Edge detection techniques, such as the Canny edge detector, have also found utility in medical imaging, particularly for emphasizing structural discontinuities indicative of fractures. While traditional edge detection algorithms are not typically integrated into deep learning pipelines, recent studies have explored their potential to enhance feature extraction in CNNs. For instance, Panda et al. [25] demonstrated that preprocessing radiographic images with edge-detection filters improved the sensitivity of fracture detection models, particularly for subtle injuries. Similarly, Bharodiya et al. [26] enhanced edge detection method for X-ray images using a combination of Gaussian filtering for image preprocessing and statistical range calculation for edge detection. The method identifies edges by calculating the difference between maximum and minimum pixel values in 3 × 3 matrix partitions.

Building upon these findings, our research combines the strengths of DenseNet121 and edge detection algorithms to create a more robust and efficient fracture detection model. By integrating a Canny edge detector into the preprocessing pipeline and introducing architectural modifications to DenseNet121, we aim to enhance the model ability to detect minor fractures with high precision while maintaining computational efficiency. This approach not only addresses the limitations of existing models but also establishes a foundation for future advancements in the automated diagnosis of sports-related injuries.

## 3. The Methodology

In this study, we present an innovative methodology for the detection of bone fractures, which are particularly prevalent in athletes. Bone fractures, medically termed as broken bones, can be precipitated by specific sporting activities and the repetitive forces exerted during actions such as running and jumping. These activities heighten the risk of certain fractures that, if not identified promptly, may necessitate surgical intervention for correction, cause in life, there are many different types of fractures; moreover, a specific fracture type can be diagnosed depending on a few criteria, as shown in Figure 1. Our novel detection model is designed to identify such injuries early, potentially circumventing the need for surgical procedures. The methodology section of our research delineates the framework of our investigation, with Section 3.1 detailing the implementation of the baseline model, DenseNet121, and Section 3.2 providing a comprehensive explanation of our proposed model.

### 3.1. DenseNet121

DenseNet121, a Dense Convolutional Network with 121 layers, represents a significant advancement in deep learning architecture due to its densely connected convolutional layers. The foundational principle of DenseNet is to maximize information flow between layers, enabling more efficient feature reuse and gradient propagation. Unlike traditional architectures, DenseNet121 establishes direct connections between each layer and every subsequent layer, ensuring that features from earlier layers are available throughout the network. This dense connectivity not only mitigates the vanishing gradient problem but also enhances feature learning with a reduced number of parameters. The architecture is structured around dense blocks and transition layers, which collectively optimize feature extraction and computational efficiency. Dense blocks are composed of layers that receive feature maps from all preceding layers as input. Each layer within a dense block applies batch normalization, a ReLU activation function, and a 3 × 3 convolution, concatenating its output with the input to maintain an enriched and cumulative feature map representation. This progressive feature aggregation ensures that critical low-level and high-level features are preserved throughout the network. Transition layers, positioned between dense blocks, serve to compress feature maps and manage the growth of feature dimensions. These layers include batch normalization, a 1 × 1 convolution to reduce the number of feature maps, and a 2 × 2 average pooling operation to downsample spatial dimensions. This design strategically balances model complexity and performance, enabling DenseNet121 to achieve superior accuracy with fewer parameters and lower computational requirements compared to architectures such as ResNet. As a result, DenseNet121 has become a preferred choice for applications demanding efficient and accurate feature extraction, including medical image analysis.

A key component of DenseNet is its growth rate *k*, which refers to the number of feature maps produced by each layer within a dense block. DenseNet121 generally uses a growth rate of 32, which means each layer adds 32 feature maps to the collective knowledge of the network, contributing to the final decision. Each layer in a Dense Block has direct access to the gradients from the loss function and the original input signal, leading to implicit deep supervision. This connectivity pattern significantly improves the efficiency of the network, making it capable of achieving high accuracy with fewer parameters compared to other deep architectures. At the end of the last dense block, a global average pooling is performed on the feature maps. This pooled output is then fed into a fully connected layer, followed by a softmax activation to produce the final classification outputs.

### 3.2. The Proposed Model

**Figure 2:**
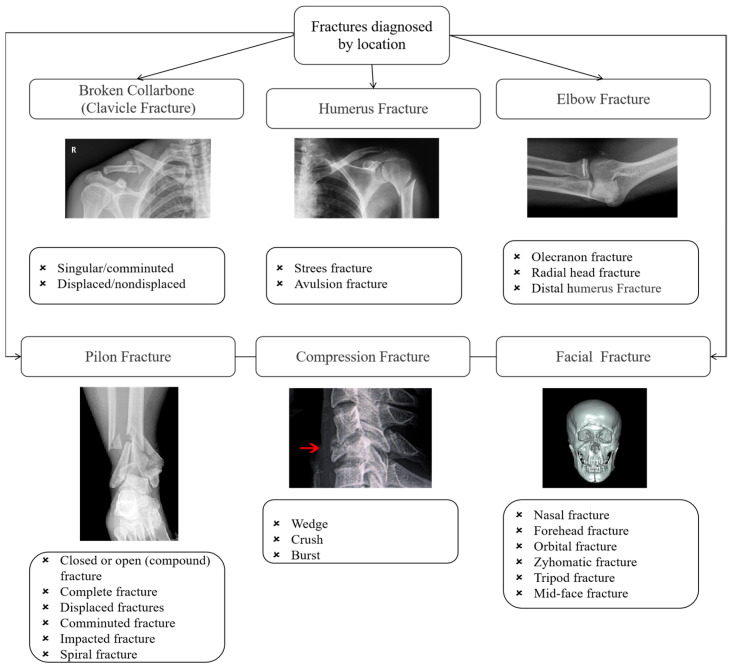
The fractures diagnosed by location.

**Figure 3:**
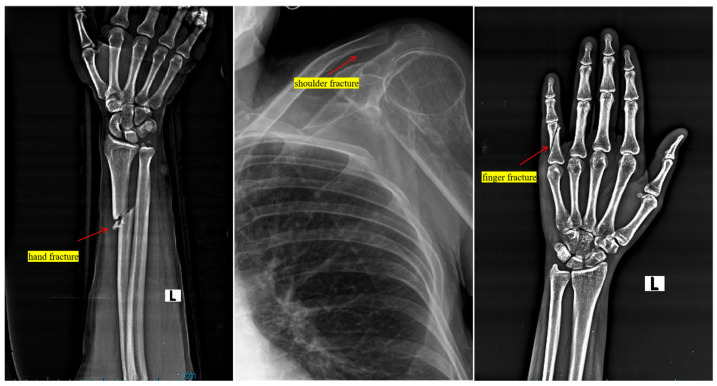
The different types of bone fractures in human body, hand, shoulder, and finger fractures.

**Figure 4:**
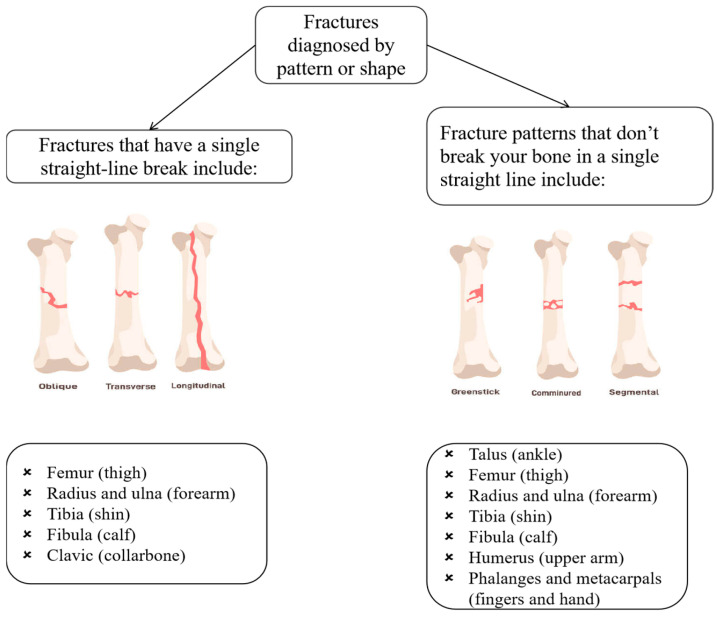
Fractures diagnosed by pattern or shape.

**Figure 5:**
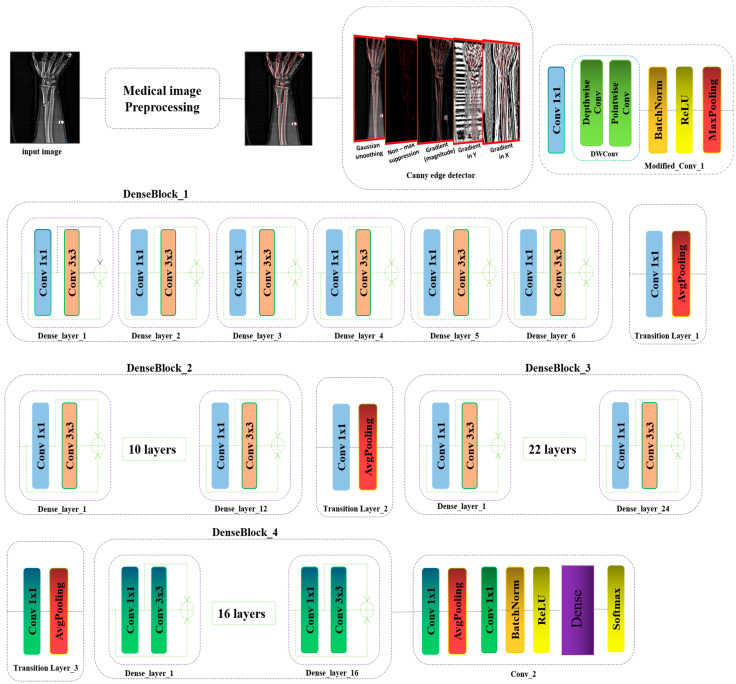
The architecture of the modified DenseNet121 for medical images.

In this study, we introduce a novel approach for detecting bone fractures in athletes, a critical factor for their health and professional longevity. We propose the integration of the Canny edge detector following the preprocessing phase, in addition to modifications to the C1 block and the final layer of our model. Bone fractures typically manifest as distinct, localized alterations in the texture and structure of bone on radiographic images. These alterations may include sharp discontinuities, atypical bone densities, or minute fragments, Figure 2, Figure 3, Figure 4 and Figure 5 Given their relatively small size compared to the overall bone structure, these features could be easily missed by a model trained on more generalized image features. By incorporating an edge detector, we enhance the ability of the model to discern more relevant features and detect even minor fractures, thereby improving its overall performance.

#### The Edge Detector

**Figure 6:**
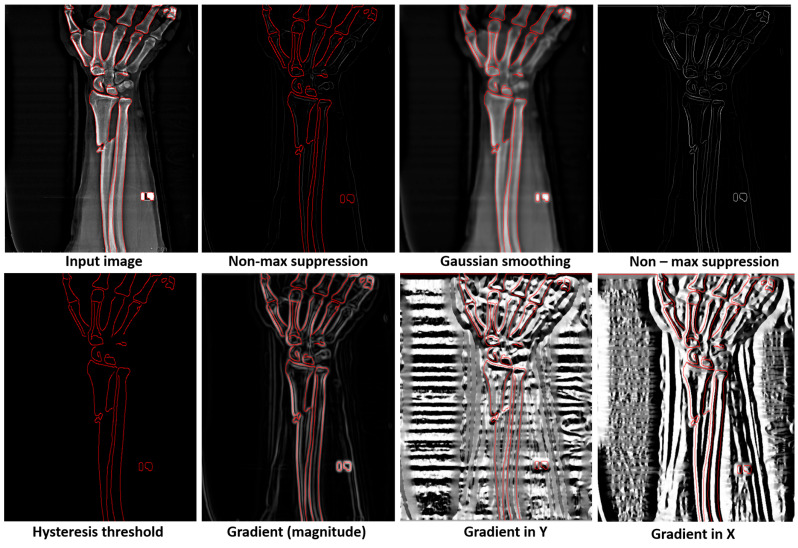
Demonstration of the 5 steps of the classical Canny edge detector with overlay and one additional grayscale non-max suppression.

The Canny edge detector, developed by John F. Canny [27], is a foundational algorithm in image processing, renowned for its accuracy and efficiency in edge detection. Its widespread application stems from its ability to identify a broad spectrum of edges while minimizing noise and false positives. The algorithm operates through a structured sequence of processes designed to enhance edge detection precision and reliability. The initial stage involves noise reduction, where a Gaussian filter is applied to smooth the image and mitigate the influence of noise, which could otherwise be falsely interpreted as edges. This preprocessing step is crucial for ensuring that the algorithm focuses on significant structural features rather than spurious details. Following noise reduction, the algorithm calculates the gradient magnitude and direction at each pixel using the Sobel operator Figure 6.

This step highlights regions of the image exhibiting strong spatial gradients, which are indicative of potential edges. To refine the detected edges further, non-maximum suppression is employed, ensuring that only the local maxima of the gradient magnitude in the direction of the gradient are retained. This process effectively thins the edges and eliminates weaker, redundant pixel responses. To classify edges, the algorithm employs a double-thresholding technique, which applies both a high and a low threshold to the gradient values. Strong edges that exceed the high threshold are immediately classified as true edges, while weaker edges are considered potential edges only if they are connected to strong edges. The final step, edge tracking by hysteresis, solidifies this classification by suppressing all weak edges that are not connected to strong edges, resulting in a clean and well-defined representation of the most prominent edges in the image. By incorporating these steps, the Canny edge detector achieves high accuracy and robustness, making it particularly effective for applications in medical imaging and other domains requiring precise feature localization. Its ability to suppress noise, enhance edge clarity, and maintain localization fidelity underscores its enduring relevance in advanced image analysis tasks.

By using this technique, we improve the accuracy of the model because, the Canny edge detector is particularly known for its accuracy; moreover, it effectively detects edges by minimizing the rate of false positives, making it suitable for the precise identification of bone fractures. Then, we reach good localization; the algorithm provides good localization of edges and it detects the edges close to the true edges in the image, which is crucial for accurately identifying the exact location of fractures in bone imagery Figure 2. Moreover, by using Gaussian filtering as a preprocessing step, the Canny edge detector is less likely to be affected by noise, making it robust for medical images, which often vary in quality and clarity. In addition, with non-maximum suppression, the Canny method ensures that the edges in the image are sharp and thin, which helps in identifying small and subtle fractures that could be indicative of early-stage injuries Figure 3.

As illustrated in Figure 4, the raw input image, denoted as $X_{raw\_img}\in R^{WxHxC}$, undergoes initial processing through the teleprocessing component tailored for medical imaging. Subsequently, the newly derived feature map is input into our newly developed edge detector. After undergoing a series of five filtering steps, the output from the edge detector, referred to as feature map $x_{Canny\_img}\in R^{WxHx1}$, is transformed into a binary image with a single channel *C* = 1. This feature map is then conveyed into the enhanced C1 block, which incorporates standard convolution, depthwise separable convolution, a normalization layer, an activation function, and pooling, as delineated in Equation (1):

(1) $$\;\;F_{Block_{1}}=F_{C1}\left(x_{Canny\_img}\right)$$

↓

$$F_{1}=F_{1\times 1}^{conv}\left(x_{Canny\_img}\right)$$

$$F_{2}=F_{PW}\left(F_{DW}\left(F_{1}\right)\right)$$

$$F_{3}=MaxPooling(max\left(0,Normalization\left(F_{2}\right)\right)$$

where, in the initial normal convolution layer, the channel count of the output from the Canny detector is modified, transforming *C* = 1 into *C* = 3. This adjustment is followed by a depthwise separable convolution layer, which captures the primary input features Figure 5. Subsequently, the normalization layer recalibrates the binary feature map, which is then processed through a max pooling operation:

(2) $$F_{Block_{2}}=F_{DBlock\_1}=F_{6}\left(F_{5}\left(F_{4}\left(F_{3}\left(F_{4}\left(F_{3}\left(F_{2}\left(F_{1}\left(F_{Block_{1}}\right)\right)\right)\right)\right)\right)\right)\right)$$

↓

$$F_{1}=F_{D\_layer1}(⊕\left(F_{Block_{1}}+F_{1\times 1}^{conv}\left(F_{Block_{1}}\right)+F_{3\times 3}^{conv}\left(F_{1\times 1}^{conv}\left(F_{Block_{1}}\right)\right)\right)$$

$$F_{2}=F_{D\_layer2}\left(⊕\left(F_{1}+F_{1\times 1}^{conv}\left(F_{1}\right)+F_{3\times 3}^{conv}\left(F_{1\times 1}^{conv}\left(F_{1}\right)\right)\right)\right)$$

$$F_{3}=F_{D\_layer3}\left(⊕\left(F_{2}+F_{1\times 1}^{conv}\left(F_{2}\right)+F_{3\times 3}^{conv}\left(F_{1\times 1}^{conv}\left(F_{2}\right)\right)\right)\right)$$

$$F_{4}=F_{D\_layer4}\left(⊕\left(F_{3}+F_{1\times 1}^{conv}\left(F_{3}\right)+F_{3\times 3}^{conv}\left(F_{1\times 1}^{conv}\left(F_{3}\right)\right)\right)\right)$$

$$F_{5}=F_{D\_layer5}\left(⊕\left(F_{4}+F_{1\times 1}^{conv}\left(F_{4}\right)+F_{3\times 3}^{conv}\left(F_{1\times 1}^{conv}\left(F_{4}\right)\right)\right)\right)$$

$$F_{6}=F_{D\_layer6}\left(⊕\left(F_{5}+F_{1\times 1}^{conv}\left(F_{5}\right)+F_{3\times 3}^{conv}\left(F_{1\times 1}^{conv}\left(F_{5}\right)\right)\right)\right)$$

Subsequently, the model progresses to the first dense block, designated as $F_{DBlock\_1}$, which comprises six internal dense layers, denoted as $F_{D\_layer1.....6}$. Each of these dense layers contains a sequence of convolutional layers: the first utilizes a kernel size of 1 × 1 and the second employs a 3 × 3 kernel. The outputs from these two convolutional layers are concatenated within each dense layer, thereby preserving information and maintaining dense connectivity throughout the network:

(3) $$F_{Block_{3}}=F_{T\_layer}=F_{1\times 1}^{conv}\left(AvgPooling\left(F_{Block_{2}}\right)\right)$$

The transition layer $F_{T\_layer1}$ is situated between two consecutive dense blocks is a transition layer that serves to compress and reduce the dimensionality of the feature maps. This layer typically encompasses batch normalization, a 1 × 1 convolution, and a 2 × 2 average pooling layer, effectively streamlining the feature maps for subsequent processing stages. After the first dense blocks comes three other dense blocks and three transition layers, as shown in Equations (4)–(8):

(4) $$F_{Block_{4}}=F_{DBlock\_2}=F_{6}\left(F_{5}\left(F_{4}\left(F_{3}\left(F_{4}\left(F_{3}\left(F_{2}\left(F_{1}\left(F_{Block_{3}}\right)\right)\right)\right)\right)\right)\right)\right)$$

(5) $$F_{Block_{5}}=F_{T\_layer}=F_{1\times 1}^{conv}\left(AvgPooling\left(F_{Block_{4}}\right)\right)$$

(6) $$F_{Block_{6}}=F_{DBlock\_3}=F_{6}\left(F_{5}\left(F_{4}\left(F_{3}\left(F_{4}\left(F_{3}\left(F_{2}\left(F_{1}\left(F_{Block_{5}}\right)\right)\right)\right)\right)\right)\right)\right)$$

(7) $$F_{Block_{7}}=F_{T\_layer}=F_{1\times 1}^{conv}\left(AvgPooling\left(F_{Block_{6}}\right)\right)$$

(8) $$F_{Block_{8}}=F_{DBlock\_4}=F_{6}\left(F_{5}\left(F_{4}\left(F_{3}\left(F_{4}\left(F_{3}\left(F_{2}\left(F_{1}\left(F_{Block_{7}}\right)\right)\right)\right)\right)\right)\right)\right)$$

The concluding segment of the architecture is identified as Conv_2, where the last transition layer is augmented with additional convolution, normalization, and an activation function. Subsequent to these layers, a dense layer equipped with a softmax activation function is employed, facilitating the final classification or prediction process:

(9) $$F_{Block9}=Softmax\left(F_{Dense}\left(max\left(0,\left(Normalization\left(F_{1\times 1}\left(AvgPooling\left(F_{1\times 1}\left(F_{Block_{8}}\right)\right)\right)\right)\right)\right)\right)\right)\;$$

## 4. The Experiment and Results

### 4.1. Dataset and Medical Image Preprocessing

In our study, a custom-curated dataset was meticulously developed to focus on the detection of fractures in athletes. The dataset includes imaging data from the most frequently injured anatomical regions, such as the elbow, fingers, hands, arms, thighs, legs, knees, shoulders, and wrists. These specific areas were chosen due to their heightened vulnerability to fractures resulting from the high-impact and repetitive physical demands of athletic activities. By concentrating on these zones, the dataset ensures relevance and practical applicability for sports-related injury detection. Before analysis, the dataset underwent a rigorous preprocessing regimen aimed at enhancing the accuracy and reliability of the fracture detection model. The images were categorized into two primary classes, labeled as ‘fracture’ and ‘no fracture’, to facilitate supervised learning. To improve the visibility of subtle features associated with fractures, contrast enhancement techniques were applied. This process emphasized slight variations in bone density, enabling the model to identify fractures more effectively.

We provide a detailed description to ensure clarity and transparency regarding the dataset utilized in this study. The dataset comprises radiographic images sourced from various sports clinics over five years. It includes approximately 5000 pictures of patients who have sustained sports-related injuries. The dataset demographics are diverse, encompassing an age range from 18 to 50 years, with a gender distribution of 60% male and 40% female participants. The included images cover a range of common sports injuries, from simple fractures to more complex cases involving multiple fracture types.

Image normalization was implemented to ensure uniformity across the dataset, scaling pixel values to a standard range. This step was crucial for stabilizing the learning process and accelerating convergence during model training. Additionally, data augmentation techniques were applied to improve the model robustness against variations in imaging conditions. Rotations, scaling transformations, and horizontal flipping were employed to expand the diversity of the dataset, enhancing the model ability to generalize to unseen images. By combining these preprocessing steps, the curated dataset provides a robust foundation for training and validating the proposed fracture detection model. This comprehensive approach ensures that the dataset captures the intricacies of athletic injuries while addressing challenges such as image variability and subtle fracture features.

### 4.2. The Used Metrics

In this research, a range of performance metrics was employed to evaluate the efficacy and efficiency of various machine-learning models designed for bone fracture detection in athletes. These metrics provide a comprehensive assessment of model performance, offering insights into their practical applicability and reliability in clinical settings. Accuracy, as a primary metric, quantifies the proportion of correct predictions, including both true positives and true negatives, relative to the total predictions made. This metric is particularly informative when the dataset classes are balanced, offering an overarching measure of model effectiveness. The number of trainable parameters (denoted as Params) serves as an indicator of model complexity. Models with fewer parameters are especially advantageous in clinical environments, where limited computational resources and the need for rapid analyses are critical considerations. A lower parameter count translates to reduced hardware costs and enhanced efficiency. Floating-point operations per second (FLOPs) provide a measure of the computational demand imposed by the model. In medical imaging applications, where prompt and accurate diagnoses are paramount, models with lower FLOPs enable faster processing while maintaining diagnostic precision. Recall, or sensitivity, measures the model ability to correctly identify all true positive cases, ensuring that no actual fractures are overlooked. This metric is crucial in medical applications, as missed diagnoses can lead to delayed treatment and adverse outcomes. Conversely, precision assesses the proportion of true positive predictions among all positive predictions, minimizing the occurrence of false positives that could result in unnecessary interventions. The F1 score offers a balanced perspective by harmonizing precision and recall into a single metric. This composite measure is particularly valuable in scenarios where both false positives and false negatives carry significant consequences, as is the case in medical diagnostics.

By leveraging these metrics, the study achieves a nuanced evaluation of model performance, balancing accuracy, efficiency, and clinical utility. This holistic approach ensures that the proposed model not only excels in detecting bone fractures with precision and reliability but also meets the operational demands of real-world medical settings.

### 4.3. Comparative Analysis of Edge Detection Techniques

We conducted a comprehensive evaluation of several widely used edge detection techniques. This section of the manuscript details the implementation and comparison of the Canny, Sobel, Prewitt, Roberts, and Scharr methods to ascertain their suitability for detecting sports-related bone fractures in radiographic images. Each edge detection technique was applied to the same dataset of radiographic images, ensuring consistency in testing conditions. The parameters for each method were carefully chosen based on standard practices in medical image processing to optimize the detection quality and to ensure a fair comparison across all methods. Table 1 presents the accuracy, sensitivity to noise, and computational efficiency of each technique. These metrics were calculated based on the aggregate results from multiple tests, providing a clear statistical overview of each method’s performance.

Table 1 summarizes the accuracy, sensitivity to noise, and computational efficiency for each edge detection technique. Accuracy is measured as the percentage of correctly identified edges corresponding to actual fracture lines. Sensitivity to noise reflects the method ability to perform under varying image qualities. Computational efficiency is measured in milliseconds, indicating the time taken to process a standard image. The performance of each edge detection technique is quantitatively summarized in Table 1. The Canny edge detector demonstrated the highest accuracy and robustness to noise, making it particularly suitable for the precise identification of fracture lines in noisy radiographic images. Computational efficiency, as shown, varies slightly across methods, with the Roberts method being the fastest, though at a slight cost to noise sensitivity.

Our analysis revealed varying levels of performance across the different edge detection techniques. While the Sobel and Prewitt methods are known for their simplicity and speed, they did not perform as well as the Canny detector in terms of accuracy and noise robustness in our specific application. The Roberts method provided sharp-edge detection but was more susceptible to noise, affecting its practical use in clinical settings. The Scharr method showed improvements over Sobel and Prewitt in handling noise but still fell short of the performance benchmarks set by the Canny detector. The Canny edge detector emerged as the most effective method, demonstrating superior accuracy and the best balance between detail sensitivity and noise suppression, which are critical for the reliable identification of subtle fracture lines in sports medicine. Given these results, the Canny edge detector was selected for integration into our diagnostic model. It provides an optimal balance between computational efficiency and diagnostic precision, essential for real-time medical applications where both speed and accuracy are crucial. The findings from this comparative study substantiate the use of the Canny edge detector in our model, enhancing the credibility and scientific rigor of our approach to automated fracture detection.

### 4.4. Comparison with Other Models

In this section, we present a detailed comparison of the proposed model with baseline and several SOTA models in bone fracture detection. The analysis focuses on metrics such as accuracy, computational efficiency, and clinical applicability, supported by [13,14,15,16,17,18,19,20,21,22].

Table 2 provides a comprehensive comparison of various state-of-the-art models on a dataset designed for detecting bone fractures, highlighting their performance across several metrics. ResNet-50, known for its robustness, achieves an accuracy of 0.872 and balances its computational efficiency with 4.85 gigaflops and 21.5 million parameters, offering solid recall and precision values. In contrast, VGG-16, despite its considerable parameter count of 128 million and higher computational demand of 13.5 gigaflops, shows lower accuracy at 0.821, which is reflected in its similar recall and precision metrics.

**Figure 7:**
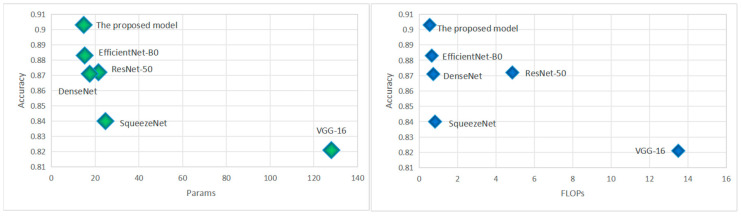
The visualization of the baseline model results.

DenseNet121 presents a competitive accuracy of 0.871 with a significantly lower computational footprint of only 0.73 gigaflops and 17.5 million parameters, maintaining high recall and slightly improved precision over ResNet-50. SqueezeNet, despite having the highest number of parameters at 24.7 million, manages an accuracy of 0.84, indicative of its less efficient design relative to its performance outputs. EfficientNet-B0 stands out with the highest accuracy of all models at 0.883, coupled with the lowest computational needs—only 0.65 gigaflops and 15.2 million parameters—resulting in the best recall and precision among the conventional models Figure 7. Finally, the proposed model surpasses all others, delivering an exemplary accuracy of 0.903 with the least computational demand of 0.54 gigaflops and 14.78 million parameters, achieving superior recall and precision, thus demonstrating its potential for efficient and accurate fracture detection in athletes.

This comparative analysis underscores the advancements in model efficiency and effectiveness, with the proposed model setting new benchmarks for performance in medical image analysis, particularly in the critical area of fracture detection.

Table 3 presents a comparative evaluation of the proposed model and various SOTA approaches in bone fracture detection, based on metrics such as accuracy, parameter count (Params), computational complexity (FLOPs), recall, and precision. The proposed model demonstrates the highest accuracy at 90.3%, significantly outperforming other models, including Saad et al. [21] (88.9%) and Kassem et al. [16] (88.3%). This superior performance highlights its capability in handling subtle and complex fracture features with precision. In terms of efficiency, the proposed model uses only 14.78 million parameters, making it far lighter than models such as VGG-16 (128 million) and Yildiz et al. [20] (22.6 million). It also achieves the lowest computational complexity, with 0.54 GFLOPs, outperforming even the efficient models like Karanam et al. [17] (0.58 GFLOPs) and Zou et al. [18] (0.69 GFLOPs). This computational efficiency ensures its suitability for real-time applications, particularly in resource-constrained clinical settings. The recall of the proposed model, at 0.89, demonstrates its exceptional sensitivity, ensuring accurate detection of fractures and minimizing missed diagnoses. Its precision, recorded at 0.875, reflects its ability to reduce false positives while maintaining high diagnostic reliability. This balance between sensitivity and specificity is crucial for medical applications.

The proposed model establishes itself as a benchmark in bone fracture detection by achieving a rare combination of high accuracy, efficiency, and clinical utility, surpassing existing SOTA models in key performance areas. This highlights its potential for transformative impact in sports medicine and broader medical imaging applications.

## 5. Discussion

In this study, we presented a novel deep learning-based framework for the detection of sports-related bone fractures, addressing critical challenges in accuracy, efficiency, and clinical applicability. Building upon the DenseNet121 architecture, we introduced architectural modifications and integrated a Canny edge detector to enhance feature extraction and sensitivity to subtle and complex fracture patterns. Comprehensive experimental evaluations demonstrated the superiority of our approach, achieving a state-of-the-art accuracy of 90.3%, coupled with exceptional computational efficiency characterized by the lowest FLOPs (0.54 G) and parameter count (14.78 M) among the models analyzed. Compared to existing state-of-the-art methods, our model excels in both sensitivity (recall: 0.89) and specificity (precision: 0.875), ensuring reliable diagnostic outcomes. The integration of preprocessing techniques such as contrast enhancement and data augmentation further improved the model robustness across varying imaging conditions. These advancements collectively address key limitations of current diagnostic approaches, including the difficulty of detecting minor fractures and the computational inefficiencies of traditional models. By achieving a rare combination of high accuracy and low computational complexity, the proposed framework sets a new benchmark for bone fracture detection in medical imaging. Its lightweight architecture and scalability make it particularly suitable for real-time clinical deployment, significantly improving diagnostic workflows in sports medicine and other domains. Furthermore, the use of a custom-curated dataset focused on athletic injuries highlights the model practical applicability to sports-related healthcare challenges. Future work will aim to expand the model capabilities by integrating additional medical imaging modalities, such as CT and MRI, to further enhance diagnostic accuracy for complex cases. Exploring advanced attention mechanisms and multi-modal learning approaches could also refine the model ability to interpret diverse fracture types and imaging conditions. By continuing to address the evolving needs of clinical practice, this research contributes to the ongoing transformation of automated diagnostics in sports medicine and beyond.

### 5.1. Ethical and Regulatory Considerations

The integration of AI in medical diagnostics presents a profound opportunity to enhance patient care and streamline clinical workflows. However, this advancement also brings forth significant ethical challenges and regulatory scrutiny. As we develop AI-driven models for detecting sports-related bone fractures, it is imperative to address these concerns comprehensively. AI systems can inadvertently perpetuate or even exacerbate biases present in their training data. Recognizing this, we have taken steps to ensure that our dataset is as representative as possible of the diverse population that may present with sports-related injuries. This includes efforts to balance the dataset concerning age, gender, and ethnicity and to include a wide range of injury types. Moreover, we continuously monitor and update our algorithms to mitigate any emergent biases and ensure fairness in diagnostic outcomes. Transparency in AI is crucial for building trust among clinicians and patients. To this end, our model is designed to provide explanations for its diagnostic decisions, making its workings interpretable to human operators.

Protecting the privacy of patient data used in training and operating our AI model is paramount. We adhere to the General Data Protection Regulation (GDPR) and other relevant privacy laws by implementing stringent data protection measures, including data anonymization and secure data storage and transmission protocols.

### 5.2. Error Analysis

Recognizing the importance of a comprehensive evaluation for our AI model, particularly in medical diagnostics where accuracy is crucial, we conducted an in-depth error analysis. This analysis focuses on instances where the model generated false positives and false negatives during its testing phase. Understanding these errors is vital for pinpointing the model weaknesses and refining its capabilities to ensure reliability in clinical settings. Our analysis highlighted that false positives often arose from images containing artifacts similar to fracture lines such as surgical screws and plates. Another common cause was the high variability in bone density, which sometimes led the model to misidentify normal anatomical features as fractures. Conversely, false negatives predominantly appeared in subtle fractures that did not significantly alter the continuity of the bone line or were masked by overlapping tissues. The error cases allowed us to discern several patterns affecting the model’s performance. Image quality emerged as a significant factor, with lower quality images leading to increased errors due to poor contrast and noise. Additionally, certain fracture types, particularly greenstick and hairline fractures, presented more challenges due to their less distinct nature.

To mitigate these issues, we propose enhancing the training dataset with more examples of subtle fractures and images containing common artifacts. This should help the model learn to distinguish better between genuine fractures and similar-looking structures. We also suggest refining the model sensitivity to subtle changes through adjustments in the feature extraction layers, which could help reduce false negatives. Addressing image quality issues through improved preprocessing techniques may also decrease the occurrence of false positives.

Our future efforts will focus on expanding the validation of the model by incorporating a broader range of data from multiple imaging centers, encompassing various fracture cases and imaging conditions. Additionally, we plan to implement a feedback loop with clinical users to gather real-time data on the model performance. This continuous monitoring will facilitate ongoing refinements, enhancing the model’s diagnostic accuracy.

## Figures and Tables

**Table 1:** Performance metrics of edge detection techniques.

Edge Detection Technique	Accuracy (%)	Sensitivity to Noise	Computational Efficiency (ms)
Canny	92	High	15
Sobel	87	Medium	10
Prewitt	86	Medium	10
Roberts	85	Low	8
Scharr	89	High	12

**Table 2:** The results of the SOTA models on bone fracture dataset.

Model	Accuracy	Params [M]	FLOPs [G]	Recall	Precision
ResNet-50	0.872	21.5	4.85	0.86	0.811
VGG-16	0.821	128	13.5	0.83	0.81
DenseNet121	0.871	17.5	0.73	0.86	0.822
SqueezeNet	0.84	24.7	0.82	0.82	0.81
EfficientNet-B0	0.883	15.2	0.65	0.87	0.853
The proposed model	0.903	14.78	0.54	0.89	0.875

**Table 3:** Comparative analysis of the proposed model performance against several SOTA models.

Model	Accuracy	Params (M)	FLOPs (G)	Recall	Precision
Lui et al. [13]	87.2%	21.5	4.85	0.86	0.811
Tieu et al. [14]	82.1%	128	13.5	0.83	0.81
Ju et al. [15]	87.1%	17.5	0.73	0.86	0.822
Kassem et al. [16]	88.3%	15.2	0.65	0.87	0.853
Karanam et al. [17]	88.1%	20.4	0.58	0.86	0.854
Zou et al. [18]	85.9	19.89	0.69	0.84	0.85
Medaramatla et al. [19]	83.7	21.4	0.89	0.83	0.84
Yıldız et al. [20]	84.7	22.6	0.67	0.84	0.83
Saad et al. [21]	88.9	25.87	0.58	0.87	0.89
Chien et al. [22]	87.59	22.13	0.69	0.87	0.88
Proposed Model	90.3%	14.78	0.54	0.89	0.875

## Data Availability

All used datasets are available online which have open access.
